# Separating the Wheat from the Chaff: RNA Editing and Selection of Translatable mRNA in Trypanosome Mitochondria

**DOI:** 10.3390/pathogens8030105

**Published:** 2019-07-18

**Authors:** Dmitri A. Maslov

**Affiliations:** Department of Molecular, Cell and Systems Biology, University of California, Riverside, CA 92521, USA; maslov@ucr.edu

**Keywords:** kinetoplast, *Leishmania*, mitochondrial translation, mitoribosome, RNA editing, *Trypanosoma*

## Abstract

In the mitochondria of trypanosomes and related kinetoplastid protists, most mRNAs undergo a long and sophisticated maturation pathway before they can be productively translated by mitochondrial ribosomes. Some of the aspects of this pathway (identity of the promotors, transcription initiation, and termination signals) remain obscure, and some (post-transcriptional modification by U-insertion/deletion, RNA editing, 3′-end maturation) have been illuminated by research during the last decades. The RNA editing creates an open reading frame for a productive translation, but the fully edited mRNA often represents a minor fraction in the pool of pre-edited and partially edited precursors. Therefore, it has been expected that the final stages of the mRNA processing generate molecular hallmarks, which allow for the efficient and selective recognition of translation-competent templates. The general contours and several important details of this process have become known only recently and represent the subject of this review.

## 1. Introduction

Trypanosomes, leishmanias, and their close relatives constitute the family Trypanosomatidae of the class Kinetoplastea [1,2]. Kinetoplastids also include a broad assemblage of organisms collectively referred to as bodonids. While trypanosomatids are obligatory parasitic, bodonids display a wide range of adaptations and are hypothesized to have undergone several independent transitions from the free-living to the parasitic life [3,4,5]. The class is primarily defined by the presence of the kinetoplast—an organelle originally believed to be involved in cell motility due to its proximity to the flagellar basal body [6,7]. The mitochondrial nature of this organelle has become clear after investigation of its ultrastructure by electron microscopy [8]. As would turn out later, the mitochondrial genome of kinetoplastids is one of the most unusual among those studied, in line with many other properties of these organisms (recently reviewed in [9]). Over the last few decades, most attention has been paid to the pathogenic trypanosomatids, and only recently has it become clear that the rest of the family is rather diverse [10]. One of the best studied pathogens is *Trypanosoma brucei*, which represents a group of several closely related subspecies implicated in devastating African diseases, such as “sleeping sickness” in humans and “nagana” in cattle [11]. These parasites propagate in the bloodstream of an infected host by utilizing an ingenious system of surface antigen switching, which allows them to avoid elimination by the host’s immune response [12,13]. The trypanosome’s life cycle also includes specific interactions with the vector, tsetse flies [14]. Importantly, mitochondrial activity is upregulated in the vector and downregulated in the bloodstream [15]. Humans are innately immune to one particular subspecies (*T. b. brucei*) [16], which is widely used as a laboratory model for this group of pathogens. Another well studied trypanosomatid, *Leishmania tarentolae*, represents another group of parasites, which cause diseases collectively affecting millions of people in tropical and subtropical regions worldwide [17,18]. The pathogenic leishmanias are transmitted by sand flies and develop chronic or lasting infections by propagating inside the host’s macrophages. *L. tarentolae* is not pathogenic to humans, being a member of the subgenus *L. Sauroleishmania*, which comprises parasites of reptiles [19]. However, a close phylogenetic relationship of the sauroleishmanias with typical human pathogens, such as *Leishmania mexicana* or *Leishmania donovani* [20], allows for an adoption of the former as a laboratory model. The best studied trypanosomatids also include *Crithidia fasciculata*, a representative of the large group restricted to parasitizing insects [21,22], and *Phytomonas serpens,* one of the trypanosomatid species that parasitize plants [23,24]. The ecological species- or strain-specific differences notwithstanding, most studied trypanosomatids have shown a remarkable conservation in the general organization and expression of their mitochondrial genetic system [25]. The choice of the study organism is often dictated by practical considerations, such as the ease of cultivation (the case of *C. fasciculata* and *L. tarentolae*) and availability of the advanced RNAi-based and other reverse genetics tools (the case of *T. brucei*), or by specific study aims, such as adaptations to plant parasitism (*P. serpens*) or developmental gene regulation (*T. brucei*). On the other hand, bodonids, which are highly significant ecologically [26], are more diverse than trypanosomatids with regard to their mitochondrial genome organization but so far remain studied only sporadically [27,28].

## 2. Kinetoplast DNA and the Discovery of mRNA Editing

Typically, as seen in trypanosomes and their close relatives, all mitochondrial DNA is localized in a particular area of the single mitochondrion of a cell [29]. This area, termed the kinetoplast, is positioned close to the basal body of the flagellum to which it is connected by a filamentous structure [30]. The mitochondrial (kinetoplast) DNA (kDNA) is organized in situ as a dense aggregation of two types of catenated DNA circles: The maxicircles and the minicircles [31]. The maxicircles (20–40 kb, the size being species-specific) represent the equivalent of mitochondrial DNA as typically seen in other organisms, whereas the minicircles (1–10 kb) are quite unique, as discussed below. The maxicircles contain two ribosomal RNA genes: The 9S small subunit (SSU) and the 12S large subunit (LSU) rRNAs, and 18 protein-coding genes, most of which represent components of the mitochondrial respiratory chain, such as three subunits of cytochrome *c* oxidase (COI–COIII), a subunit of cytochrome *bc*_1_ (apocytochrome *b* or Cyb), and several subunits of NADH dehydrogenase (ND1–ND9) [32,33,34]. There are also two genes for ribosomal proteins: S12 (RPS12, former G6 or CR6) [35] and S3 (former maxicircle unidentified reading frame 5 or MURF5) [36]. There are no tRNA genes in the kDNA, with the mitochondrial translation system utilizing the imported cytoplasmic tRNAs [37,38]. The coding region is compact (~17 kb), with the gene order and the strandedness being generally conserved among the species [39]. The rest of the maxicircle is occupied by the ‘divergent region’ composed of numerous repeats of an unknown function [40,41,42]. Twelve maxicircle genes demonstrate encoded defects of varying magnitude: From small frameshifts to unrecognizable gene regions or even entire genes (the latter termed ’cryptogenes’) [43,44]. In the seminal discovery of RNA editing, Benne and colleagues demonstrated that a frameshift in the COII reading frame is repaired by post-transcriptional insertion of four U-residues in the mRNA [45]. The subsequent analyses of other maxicircle genes showed that the U-insertions or, less frequently, deletions of the encoded Us by RNA editing can generate the missed initiation and termination codons, repair the internal frame-shifts, restore the missed 5′-coding regions, and, in extreme cases, create an entire reading frame out of the encoded GA-skeleton [46,47]. In the latter case, the amount of editing required is staggering, with the fully edited product nearly doubling the size of the pre-edited mRNA (’pan-editing’) [48,49]. In some species, e.g., in *T. brucei*, a larger fraction of genes exists as cryptogenes, while in others, e. g. in *L. tarentolae* and other leishmanias, the so-called ‘5′-edited’ or ‘internally edited’ versions are more common, entailing a reduction in the overall editing required [50,51,52]. It was hypothesized that in the course of evolution, the ancestral pan-edited cryptogenes were partially replaced with their less-edited counterparts [53].

Obviously, the process of editing depends on a massive and orderly flow of genetic information originating from a source within the mitochondrion. The search for its depository led to the discovery of guide RNA (gRNA) genes, first in the maxicircles [54] and shortly thereafter in the minicircles, where most of these genes turned out to be located [55,56]. This finding provided a solution to the long-standing conundrum regarding the genetic role of these small DNA molecules, thousands of which form the bulk of the kDNA. Each individual minicircle molecule encodes from one to four gRNAs, depending on the species [57,58,59]. According to the coding capacity, the entire minicircle population within a kDNA network is subdivided into multiple classes, which collectively provide dozens or even hundreds of different gRNA types necessary for editing. The minicircle repertoire varies depending on species: It is substantially larger in *T. brucei* compared to the *Leishmania* species in accordance to a higher prevalence of pan-editing in trypanosomes [60,61].

Regardless of their origin from minicircles or maxicircles, most gRNAs have the same plan of sequence organization. These 30 to 50 nt transcripts are complementary (also allowing for G-U base pairing) to the edited segments of mRNAs and contain a post-transcriptionally added 3′ oligo(U)-tail [62]. According to the original insightful model, the gRNA is directed to the cognate pre-edited region in the mRNA by the complementarity between the first 10 to 12 nt at the 5′ end of the gRNA and the equivalent segment just 3′ of the first editing site in the mRNA [54]. Following the ‘anchor duplex’ formation, the mismatches between gRNA and mRNA will guide a series of U-insertions and deletions in the latter until both molecules become fully complementary. It was proposed that editing involves the ‘enzymatic cascade’ reactions, composed of an endonucleolytic cleavage at the site of a mismatch, U-insertions by terminal uridylyl transferase (TUTase) or U-deletions by exonuclease, and a re-ligation [54]. The mismatches were originally thought to be recognized in a strictly sequential 3′-to-5′ fashion beginning from the anchor duplex but the process may actually proceed less orderly [63,64,65,66,67]. The interactions between the gRNA’s 3′ oligo(U)-tail and the mRNA’s G/A-rich pre-edited region help to keep the mRNA segments in place after cleavage [62].

## 3. Enzymology of the Core RNA Editing Reactions

Intensive studies of the enzymes and auxiliary factors involved in RNA editing revealed an unprecedented complexity of this process. This topic has been covered by several excellent reviews [68,69,70,71,72] and is discussed here only briefly. The basic set of reactions is performed by 20S RNA editing core complex (RECC), which has a modular structure and is composed of 20 polypeptides [73,74]. The three structural modules of RECC represent three distinct editing reaction cascades: U-insertions, U-deletions, and a peculiar kind of U-insertions into COII mRNA (which is performed by a unique *cis*-acting gRNA residing at the 3′ untranslated region of the COII mRNA), with each module defined by an associated endonuclease [75,76]. These RNAse III type enzymes cleave the mRNA at the mismatch site: KREN1 cleaves at the deletion sites, KREN2 cleaves at the insertion sites, and KREN3 cleaves at the *cis*-edited insertion sites in COII mRNA. Each endonuclease forms a heterodimer with a respective catalytically inactive auxiliary protein. The KREN1 deletion module also contains KREX1 exonuclease to trim the mismatching U-residues. The modules associate with the common structural core of the complex on the mutually exclusive basis by docking to the respective binding sites. Thus, the KREN1 deletion module binds to the core’s deletion compartment (subcomplex), which contains KREL1 ligase, a second (and dispensable) exonuclease KREX2, and an auxiliary protein [77,78]. The KREN2 insertion module associates with a different compartment (subcomplex) of the core: This subcomplex contains KRET2 terminal uridylyl transferase, which adds U-residues at the 3′-end of the cleaved segment of mRNA, a specialized KREL2 ligase (also dispensable), and an auxiliary protein [79,80]. The core also contains the ‘inner nucleus’ of four OB-fold and two zinc-finger proteins participating in the proper docking of the modules and maintaining the RECC structure.

## 4. The Dynamics of mRNA-gRNA Interactions

Obviously, a single gRNA can process only a short segment of an mRNA (termed ‘editing block’). This is sufficient to cover a short pre-edited region of several nucleotides, but a long pan-edited mRNA requires a sequential action of several gRNAs. The first gRNA initiates the process by annealing to the encoded anchor sequence close to the mRNA’s 3′ end [54,81]. Interestingly, the anchor sequence for the second gRNA is created by editing the last few sites within the first block. The process continues in a similar fashion, with the anchor for each subsequent gRNA created by the action of a preceding gRNA [82]. The editing thus spreads in the 3′-to-5′ direction along the entire mRNA until it stops approximately 10 to 20 nt before the 5′ end. In *Leishmania*, the edited anchors are normally located at the very ends of the preceding blocks ensuing the minimal overlap between consecutive gRNAs [82]. There is usually a single gRNA (two at most) for the editing of any particular mRNA region [83,84]. A loss of such indispensable gRNAs due to random loss of the respective minicircle classes has been documented in an old laboratory strain of *Leishmania* [85]. Such a loss entails a disruption of the editing cascade and the lack of the respective fully edited mRNA species. On the other hand, in *T. brucei*, the diversity of gRNA is much greater, approaching several hundred, with an overlap between consecutive gRNAs being larger due to multiple redundant gRNAs [60,64,65,66,67,86].

The process of editing is relatively slow for long pan-edited transcripts, and the steady-state populations of such mRNAs are highly heterogeneous. The final pan-edited mRNA may represent a minor fraction in the population, reflecting the net drop in efficiency with each consecutive step [65,67]. In addition, there are abundant molecules with various degrees of editing. These partially edited molecules usually contain a fully edited segment on the 3′ and remain pre-edited on the 5′ end. These two regions often are separated by a “junction” region, which shows an edited sequence that is different from the final edited sequence [87]. Editing by a non-cognate gRNA following a spurious anchor formation was proposed as the cause of junction regions in *L. tarentolae* [88,89]. This ‘mis-editing by mis-guiding’ scenario is opposed by a ‘dynamic interaction’ model, which suggests that junction sequences represent true intermediates of the process [63,65,66,67]. The latter model was derived for *T. brucei*, the organism with a more diverse minicircle and gRNA composition. It is possible that both scenarios actually take place, although with a different prevalence in the two species, as some junction regions differ only slightly from the mature sequence (the likely intermediates) and some regions matching the non-cognate guides (the likely mis-edited sequences) [67,90]. Regardless of the origin, a junction region must be re-edited to match a fully edited pattern in order to allow for the annealing of a next cognate mRNA and extension of the editing further upstream. In the investigated strains of *L. tarentolae*, which were shown to have lost several indispensable minicircle classes during the prolonged cultivation (and putatively also in certain strains of *Phytomonas serpens*, *L. donovani,* and *C. fasciculata*), the editing of several mRNAs could not be completed [85,91,92,93]. Partially edited intermediates with junction regions would then accumulate in the steady state mRNA population.

However, the re-editing requirement cannot be met for junctions that occur at the very 5′ end where the editing stops. Without the re-editing to match a canonic editing pattern, these regions would appear as alternatively edited sequences. A start codon, if present in such a transcript, would direct translation of an alternative reading frame (ARF) [94]. In some cases, the 5′ junctions have been attributed to abundant non-canonical gRNAs, and such alternatively edited transcripts could represent a sizeable fraction in a total mRNA population [65]. These observations and also the earlier findings of a second reading frame within some canonic edited sequences (e.g., edited RPS12 [95] and ND6 (CR4) [96] in *T. brucei*, and edited ND4L (G3) in *P. serpens* [97]) led to the “dual coding mRNAs” hypothesis [94]. In a few cases, a short alternative editing pattern was found in the center part or at the 3′-end of the fully edited sequences, which were canonic otherwise [98,99]. However, the alternative editing hypothesis still needs to be supported by protein-derived amino acid sequence data. The same applies to partially edited transcripts that contain an ORF within the pre-edited segment of a partially edited mRNA in-frame with the canonical edited ORF downstream [100].

The proportion of a fully edited mRNA within the total mRNA population varies depending on the species, strain, gene, and, in *T. brucei*, on the life cycle stage of the parasite. In the latter case, as exemplified by Cyb mRNA, the fully edited species abounds in the procyclic form (PF, an actively respiring insect stage), and is undetectable in the bloodstream form of parasites (BF, a ‘slender’ bloodstream form, with the downregulated mitochondrial respiratory chain) [101]. Conversely, the pan-edited mRNAs for several subunits of NADH dehydrogenase, although detectable in both stages, are more abundant in BF cells [96,102]. The mechanisms controlling the steady state levels of edited mRNAs are unknown, but it has been proposed that developmental regulation in general is not due to the differential gRNA presence/absence [66,86,103]. Nevertheless, some key gRNAs may be important as these levels correlate with a relative abundance of the first RNA in the editing cascade [66].

It is important to mention that, although editing is developmentally regulated, the correlation of the abundance of functionally edited transcripts with the anticipated pattern of mitochondrial gene expression is by far not perfect. For example, the edited COIII, which is not required during the bloodstream stage, is nonetheless present in BF parasites [48]. These considerations gave an early indication that other mechanisms may define which mRNAs are expressed in a given stage of the life cycle.

## 5. The Auxiliary Factors Enabling gRNA–mRNA Interactions

The RECC complex, capable of executing only the basic enzymatic reactions, requires the aid of an elaborate auxiliary machinery in order to perform numerous insertions and deletions in a diverse range of mRNA substrates [68,70]. This machinery was termed RNA editing substrate binding complex (RESC) or mitochondrial RNA binding complex 1 (MRB1). Its contacts with RECC are RNA-based [104], reflecting the fact that the RNA editing substrates (gRNAs and pre-edited mRNAs) and the products (partially and fully edited mRNAs) directly associate with RESC [105,106]. Guide RNAs are provided to RESC in the form of a tetrameric complex of two proteins, GAP1 and GAP2, which are also necessary for gRNA stabilization [107,108,109]. Together with a set of five additional proteins, these factors form the guide RNA binding complex (GRBC), which is regarded as a subcomplex of RESC. It is currently held that the GRBC subcomplex is required for initiation of the editing process [105,108,110]. This subcomplex interacts with REMC (RNA editing mediator complex), which represents a subcomplex necessary for the processivity of editing by facilitating recruitment of RECC to the mRNA–gRNA duplex [111,112]. REMC is composed of an RNA binding protein, RGG2, and several additional proteins [105,113].

## 6. PAMC and Additional Pre- and Post-Editing Modifications in mRNA

The third subcomplex of RESC is a group of proteins mediating interactions with the polyadenylation machinery—the polyadenylation mediator complex (PAMC). A pre-edited mRNA acquires a short (~20 nt) poly(A)-tail by the action of KPAP1 poly(A)-polymerase. This tail remains throughout the editing process and is indispensable for the mRNA’s stability past the first few editing events: Ablation of the short tail formation leads to a great reduction in the steady-state level of the partially edited mRNA but does not affect the pre-edited transcripts [114,115]. Upon completion of editing, the existing short tail is extended to become a long (200–300 nt) poly(A/U)-tail by a combined action of KPAP1 polymerase, KRET1 uridylyl transferase, and two auxiliary factors: KPAF1 and KPAF2 [116]. The current model holds that the dependence of partially edited mRNAs on the presence of a short poly(A)-tail for stability serves as a quality control mechanism to ascertain that only fully edited mRNAs will get an extended tail [68]. This coordination of editing and polyadenylation is made possible through the physical contacts which have been demonstrated for the components of PAMC and the system of polyadenylation [105].

## 7. Pentatricopeptide Proteins as Specific mRNA Recognition Factors

The fact that a translation-competent fully edited mRNA represents only a small fraction within a highly diverse pool of transcripts poses the problem of separating “the wheat from the chaff”: How are these templates selectively recognized and forwarded for translation? The answer to this question is not fully available yet, but recent works have provided some insights [117].

The processes of mRNA maturation and translation in organelles are mediated by pentatricopeptide and related tetratricopeptide repeat proteins (PPR and TPR, respectively) [118,119]. Most abundant in plants, these sequence-specific RNA-binding proteins perform a diverse set of functions in RNA splicing, editing, translation, and stability [120,121,122,123]. A PPR motif represents a variable 35 amino acid sequence arranged as two antiparallel α-helices. There has been a significant progress lately towards elucidating the rules of sequence recognition by PPR motifs [124,125,126,127]. The amino acids residues at positions 5 and 35 of a repeat recognize a certain base in RNA, and a specific combination of repeats would recognize a respective RNA sequence. The PPR protein bound to this sequence would then serve as an adaptor for recruiting necessary enzymatic or structural components to the binding site.

## 8. PPR Proteins as Modulators in mRNA Maturation

In trypanosomatids, there is an unusually high (for non-plants) number of PPR proteins—nearly 40, most of which have been demonstrated or predicted to localize in the kinetoplast-mitochondrion [117,128,129]. A score of the PPR proteins was found in association with the mitochondrial ribosomes [129,130,131,132] and the polyadenylation complex [116], which suggests their involvement in translation and 3′-end maturation, respectively. One of the ribosome-associated proteins, KRIPP11, was also found to recognize and bind to oligo(G)-tracts in RNA, as well to G-quadruplex structures [133]. The latter are formed by two or more stacked G-tetrads in which four guanine bases are held together by Hoogsteen hydrogen bonds [134]. Both G-tracts and G-quadruplexes are typical for pre-edited mRNAs which are intrinsically G-rich [135]. This recognition property would confer to the ribosome the ability to interact with pre-edited mRNA, although the functions of such interactions still remain a matter of speculation [133]. One additional PPR protein (TbPPR9) was found to be associated with a yet uncharacterized complex with some role in mRNA stabilization [136].

The functions of some kinetoplast PPR proteins participating in the pre-editing and post-editing RNA processing events have recently been determined in more detail. Contrary to the previously held notion of maxicircle transcription being polycistronic, with individual mRNAs generated by endonucleolytic processing [137], it has been shown that transcription of maxicircle genes is monocistronic [138]. The 5′-ends of mRNAs are defined by the sites of transcription initiation, while the 3′-ends are produced by a 3′ to 5′ processing of the initial 3′-extensions. This trimming is performed by mitochondrial 3′-processome (MPsome) composed of DSS1 3′–5′ exonuclease, RET1 TUTase (RNA editing terminal uridylyl transferase 1), and three additional proteins [139,140,141]. The extent of trimming is controlled by an antisense mechanism, similar to the one proposed for the 3′-end processing of minicircle-encoded gRNAs, wherein the 5′-end of the antisense transcript causes the MPsome to pause at a short distance downstream from the duplex region [140]. The PPR protein, KPAF3 (kinetoplast polyadenylation factor 3), binds to a G-rich octamers that localizes in the 3′-region of a pre-edited mRNA [142]. This recognition and binding are crucial for determining the fate on the transcript: in its absence, the transcript would become polyuridylated, as occurs with ribosomal RNA and gRNA transcripts which lack such *cis*-elements, and then further trimmed by the MPsome [140,141,143]. In the presence of a G-octamer, the bound KPAF3 would stimulate KPAP1 to add a short poly(A)-tail to the pre-edited mRNA’s 3′-end. This structure becomes crucial for the stability of the mRNAs past the first few editing events at the 3′-end, which also result in a displacement of KPAF3 [114,142]. Another PPR protein, KAPF4, has recently been demonstrated to act as poly(A)-binding protein (PABP) and also interact with polyadenylation machinery [144]. Moreover, KPAF4 bound to the poly(A)-tail has been shown to interact with RESC (via its PAMC module), and, interestingly, with the mRNA’s 5′-end, which is “capped” by a protein complex termed PPsome (5′-pyrophosphohydrolase complex). The latter complex is composed of an NUDIX hydrolase, a PPR protein, and an additional protein. The initial 5′-triphosphate at the transcription initiation site is converted to monophosphate by the action of PPsome, which must be induced by RESC to become active [138]. The current model holds that poly(A)-bound KPAF4 mediates a circularization of mRNA, which brings RESC in contact with PPsome. This multipartite interaction serves to ensure that only mRNA transcripts with the properly processed 5′- and 3′-ends would undergo editing [144].

## 9. Formation and the Role of 3′ Poly(A/U)-Tails in mRNA

There seems to be an additional quality control stage after the completion of editing. It has been hypothesized that a yet unidentified PPR protein binds the new edited sequence motif created by the last editing events at the 5′-end of the mRNA. These proteins can reach the mRNA’s 3′-end via mRNA circularization. This interaction in turn serves to recruit two additional PPR proteins, KPAF1 and KPAF2, to the 3′-end [114,116]. It was demonstrated that these factors modulate the KPAP1 poly(A)-polymerase and RET1 TUTase activities to synthesize the long (200–300 nt) poly(A/U)-extension of the pre-existing short poly(A)-tail [116]. The fact that edited mRNAs have two types of the poly(A)-tails, while pre-edited and partially edited transcripts have the single short type, was discovered decades ago [145], but the functional significance of these differences remained unknown. Recent works have also demonstrated that the long-tailed mRNA, as opposed to the short-tailed, are associated with translating ribosomes, and that ablation of the long poly(A/U)-tailing inhibits translation [116,132,146,147]. This makes the long poly(A/U)-tails the primary determinants of translationally competent mRNAs. This additional step in a rather complex system of mRNA maturation serves to make sure that only fully edited mRNAs, as opposed to partially edited transcripts, would receive the long A/U-extensions and be forwarded for translation. Interestingly, the mRNAs with an encoded ORF (such as COI), which do not require editing (‘unedited’ mRNAs), are translation-competent and, accordingly, have two types of 3′-end tails. Moreover, in *T. brucei*, the long tail formation is developmentally regulated [148], and the presence/absence of the long tail strongly correlates with the expected gene expression during the life cycle [66]. Thus, the long tailed mRNAs for subunits of mitochondrial respiratory complexes III (Cyb) and IV (COI–COIII) are found only in PF trypanosomes, which possess a fully developed mitochondrial electron transport chain, as opposed to BF trypanosomes (the inducible expression), while the RPS12 and subunit 6 of F_1_F_O_ ATPase gene products are necessary in both PF and BF trypanosomes and the respective long tailed mRNAs are detectable in both (the constitutive expression) [149,150,151].

## 10. The Initiation Codon Recognition Problem

The localization of the translatability determinants on the mRNA’s 3′-end goes in hand with their apparent absence on the 5′-end. The mechanism of initiation codon recognition remains unclear. There is no identifiable equivalent of the Shine-Dalgarno sequence. The canonical initiation codon may be preceded by an upstream AUG codon positioned in-frame or out of frame. The initiation AUG may [49,50,51,95,99,102] or may not [52,83,152] be created by editing, and in some cases, an alternative initiation codon (e.g., AUU, AUA) appears to be used [96,99,153]. In any case, the simplest scenario, which is based on utilization of the 5′-most AUG, as in mammalian mitochondria [154], does not operate in trypanosomes. On the other hand, the yeast model, which is based on a long 5′-UTR containing the *cis*-elements recognizable by a membrane-bound activator complex [155], is not applicable either because the 5′ untranslated regions (UTR) in trypanosomatids are relatively short (30–40 nt). The case is further compounded by potentially dual coding genes which contain alternative reading frames [94]. Apparently, initiation of translation in trypanosomatids proceeds by a mechanism substantially different from other systems. Considering interactions between the mRNA’s 5′ and 3′ ends during editing and a long poly(A/U)-tail formation [142,144], and also interactions between a poly(A/U)-tail and a small ribosomal subunit [116], the translation initiation likely occurs in a circularized mRNA template.

## 11. Organization of the Mitochondrial Translation Apparatus

Integration of mRNA editing/maturation processes with mitochondrial translation is further supported by the finding of a discrete in situ localization of the ribosomal subunit markers (mitochondrial riboprotein S17 for SSU and L3 for LSU) in proximity to the RNA editing complexes [156,157]. Each subunit was localized in the two antipodal nodes which were close to but distinct from the nodes of the GRBC complexes. All types of nodes were positioned close to the kDNA disk. Hypothetically, the ribosomal subunit nodes represent the sites of mRNA recognition and ribosome assembly. However, mitochondrial protein synthesis is expected to take place on the inner mitochondrial membrane since most maxicircle gene products are highly hydrophobic polypeptides [158,159]. Therefore, the translation may be restricted to the membranes surrounding the kDNA as this scenario would involve only a short distance relocation of ribosomes from the nodes.

Since the ribosomal markers were detected only within the nodes, it can be expected that the steady state level of the assembled membrane-bound ribosomes is low. This view is consistent with the biochemical data available so far and may explain why, unlike other mitochondrial ribosomes, the trypanosomatid mitoribosomes remained elusive for decades despite an abundance of the 9S and 12S rRNA [160,161]. The population of mitochondrial ribosomal RNP complexes in mitochondrial lysates from *L. tarentolae* was found to be mostly comprised by the ribosomal subunits [162]. Particles with a size (50S) and morphology reminiscent of bacterial ribosomes and the 1:1 stoichiometric ratio of the two ribosomal RNAs were also found but represented only a minor component [162]. These putative mitoribosomes were sufficiently stable to be partially purified and characterized by cryoelectron microscopy [163]. The structural map obtained at the ~14 Å resolution showed a particle with the typical ribosomal morphology. These ribosomes were ~245 Å in diameter, which is smaller than eubacterial 70S ribosomes, and substantially smaller than mammalian 55S mitoribosomes [164]. The 50S monosome was composed of 28S SSU and 40S LSU. The SSU showed a set of characteristic features, such as head, platform, shoulder, body, and spur, but was highly “porous”. The 9S SSU rRNA (610 nt) is among the smallest of its kind [165,166]. The stem-loop structures present in the eubacterial SSU rRNA, but absent in the *Leishmania* 9S rRNA, were only partially substituted with protein masses in the 50S ribosome, leaving open gaps in the head and the body [163]. The decoding center is formed with the participation of a conserved, although reduced, rRNA helix 44 (using eubacterial nomenclature) and a protein mass putatively identifiable as mitochondrially encoded PRS12. Several additional proteins with eubacterial homology, which had been identified by mass spectrometry [130,131], were also recognizable in the cryoelectron microscopy structure. The mRNA path is lined with trypanosomatid-specific proteins and the mRNA entrance is relatively wide [163], possibly reflecting the unique way of mRNA recognition by these ribosomes. The LSU also retains a characteristic morphology showing central protuberance (CP), two stalks, and body despite the drastic reductions of the size of the 12S LSU rRNA and the protein composition, which is different from the eubacterial counterpart. Thus, CP is formed with trypanosomatid-specific proteins instead of RNA as in most other systems, and so is the morphological L1 stalk (there is no identifiable eubacterial or mitochondrial L1 protein homolog in *Leishmania*). The LSU also contains open gaps due to incomplete replacement of the missing step-loop rRNA structures with proteins. The A and P tRNA binding sites are dominated by trypanosomatid-specific proteins replacing the missing LSU rRNA segments. A combination of unique and conserved features is also seen around the peptidyl transferase center and the polypeptide exit channel. Overall, the 50S structure highlighted the importance of maintaining the overall ribosomal architecture, as well as the replacement of rRNA segments with proteins as the way to accommodate the unique interactions of these ribosomes with edited mRNAs and imported cytosolic tRNAs. However, it must be mentioned that the 50S particles have not been tested for activity (such as factor binding, mRNA binding, or peptidyl transferase activity).

## 12. The Unusual SSU-Like 45S and Ribosome-Like 80S Complexes

In *L. tarentolae* and *T. brucei*, both subunits of the 50S ribosome were also found in mitochondrial lysates as free particles [132,162]. The free 40S LSU represented a major class of particles containing the 12S rRNA, while individual 28S SSU particles represented only a minor class. The most abundant among the 9S rRNA-containing particles were the distinct 45S complexes [130,132]. In addition to the 9S rRNA, these complexes contained a set of typical SSU mitoribosomal proteins (including homologs of S5, S6, S8, S9, S11, and S15–S18), several PPR proteins (dubbed as KRIPPs for kinetoplast ribosomal PPR proteins), as well as a score of additional trypanosomatid-specific proteins. These particles were termed SSU-related complexes (SSU*).

Recently, a near-atomic resolution cryo EM model of these particles was generated as part of a relatively large, ~385 Å in diameter, ribosome-like 80S complex [33]. This complex included two subunits and one of them matched the 45S SSU* by morphology and composition [130,132,162]. Two mitochondrially encoded proteins (RPS12 and MURF5, dubbed uS12m and uS3m, respectively) were identified within the SSU* along with 55 nuclear-encoded proteins, including the aforementioned SSU protein homologs and the KRIPPs. The head of this small subunit was largely expanded in comparison to typical SSUs. It was connected with the body by several protein linkages, which would make this connection less flexible and potentially hindering the necessary head movements during translation [167]. The second subunit of the 80S complexes contained the 12S rRNA and several LSU ribosomal protein homologs. This subunit was protein-rich, containing 127 proteins, and its shape appeared to be rather monolithic, especially in comparison to the 40S LSU of the 50S monosomes of *Leishmania*. In these protein-rich 80S ribosomes, the role of the structural scaffold, usually played by ribosomal RNA, has been relegated to the proteins which formed a shell encasing the particle and maintaining the two very small rRNAs in a proper functional shape. A more detailed comparison between the 80S and the 50S particles awaits the availability of a high resolution structure of the latter.

## 13. Two Types of Mitochondrial Ribosomes?

The case of two types of monosomes (50S and 80S) apparently coexisting within the same mitochondrion is puzzling. In addition, in *T. brucei*, there were ~85S particles that showed the equal stoichiometric (1:1) ratio of the 9S and 12S rRNAs [116,132]. These ~85S particles were present in a relatively low amount and were detectable only by applying a rapid analysis of a total cell lysate. A fraction of each tested long tailed mRNA species was found within the 85S complex, which was also shown to contain tRNA [116]. These data strongly suggest that the 85S complexes contain actively translating ribosomes. What are the relationships between these and the 50S or 80S complexes and what are the distinct roles (if any) of the two latter types?

The proteomics analyses confirmed that in PF cells, the 45S SSU* complexes associate with the ribosomal large subunits [116,146], apparently forming the 80S particles. The function of the 45S SSU* complexes (and by inference that of the 80S) in *T. brucei* was tested by using RNAi-based conditional knock-down of its several proteins [132,146]. The subunit was essential for the viability of PF cells, in which its selective ablation affected the stability and translation of some (COI, Cyb) long poly(A/U)-tailed mRNAs without affecting the other (RPS12, A6). Accordingly, the RNAi ablation of the 45S complexes disrupted the formation of the 85S complexes for the long-tailed COI and Cyb mRNAs but not for the RPS12 and A6 mRNAs [132,146]. These results indicate that the translation apparatus, which is engaged in translation of the RPS12 and A6 long-tailed mRNAs, does not include the 45S SSU* subunit. Moreover, out of the two tested KRIPP components of the 45S SSU* (KRIPP1 and KRIPP8), only KRIPP1 was also found within the 85S active ribosomal complexes [146]. This fact, and also the aforementioned resilience of the RPS12- and A6-translating ribosomes to the ablation of the 45S SSU* complex, also argues against this subunit (and by the same token the 80S monosome) being part of the translating 85S ribosomal complexes. The question if the latter role is played by the 50S ribosomes instead remains open. The size increase of the 85S complexes compared to the 50S could be due to the joining of additional proteins or due to a specific polysomal arrangement of the 50S monosomes, such as that described in yeast mitochondria [168].

It is important to notice that COI and Cyb mRNA are expressed only in actively respiring PF cells (the inducible expression), while RPS12 and A6 are expressed in both PF and BF trypanosomes (the constitutive expression). In line with the importance of the 45S SSU* complexes for translation of inducible (as opposed to constitutive) mRNAs in PF cells, the 45S complexes were down-regulated in BF trypanosomes [132,147]. Moreover, according to protein network analyses, these complexes were not associated with the ribosomal LSU in BF trypanosomes, and their ablation by RNAi was inconsequential for BF cell viability [132,146]. There results can be interpreted as indicating that 80S ribosomes are not formed in BF cells. Yet, the functional ribosomes were indeed present in BF cells, as shown by the proteomics analysis, which showed the association between riboprotein S17 (the SSU marker) and riboprotein L3 (the LSU marker) [164], as well as by the presence of the 85S complexes containing long-tailed RPS12 and A6 mRNAs (but not the COI mRNA) in BF cells [147]. Moreover, a direct analysis of the total BF cell lysate showed that the 50S ribosomal particles were present, although in a lower amount compared to PF trypanosomes, while the 80S particles could not be observed [132]. Thus, the data seem to argue that the 50S ribosomes (or a similar type), and not the 80S, play a functional role in BF cells, while both types are important in PF cells.

**Figure 1:**
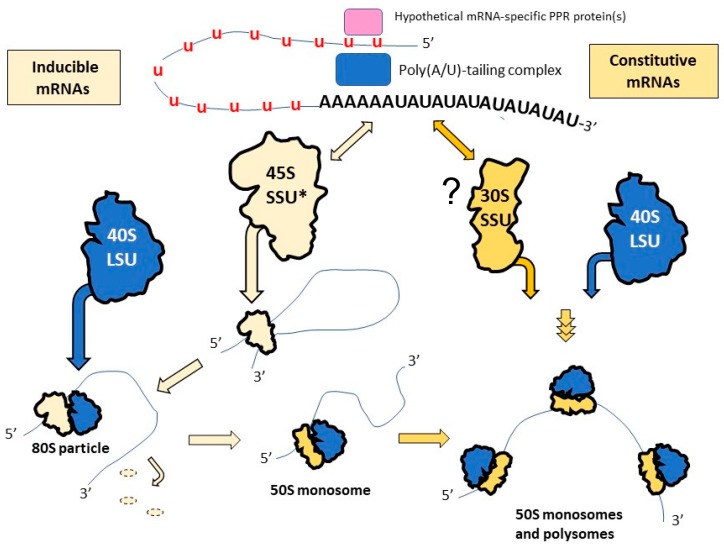
A hypothetical scenario of the roles of the 50S and 80S complexes in the formation of translating ribosomes in *T. brucei* mitochondria. Upon completion of editing at the 5′-end, a hypothetical PPR protein recruits a poly(A/U)-tail formation complex to the pre-existing 3′ oligo(A)-tail. The (A/U)-tailed mRNA is selectively recognized by mitochondrial ribosomes and translated [116,142,144]. It is hypothesized that the inducibly expressed mRNAs (such as COI and Cyb) are selectively recognized by 45S SSU*. After the joining of the 40S LSU and remodeling of the 45S SSU*, the mRNA would be translated by the 50S ribosomes. Recognition of the constitutively expressed mRNAs (A6, RPS12) would not involve the 45S SSU* complex and would proceed differently. u—uridylates symbolizing edited mRNA sequences.

The following hypothetical scenario can explain the observed properties of the mitochondrial translation apparatus in trypanosomes (Figure 1). The 45S SSU* subunit specializes in recognition of the inducibly translated mRNAs in PF cells, such as those for subunits of the respiratory complex IV (COI–COIII), complex III (Cyb), and, perhaps, at least some subunits of complex I. The recognition of the respective fully edited long tailed mRNAs is facilitated by KRIPP proteins of this large complex and involves interactions with components of the polyadenylation machinery. The initiation complex formation is followed by the joining of the LSU to form the 80S particle. A remodeling of the small subunit would follow, which includes shedding of the mRNA recognition modules and optimization of the structure for elongation (e.g., enabling rotation of the subunit’s head). Thus, a more conventional ribosome, such as the 50S particles, would constitute the translating 85S complexes which may be polysomal. The initiation of the constitutive mRNAs follows a yet unclear path which does not involve the 45S SSU* subunits but would, nonetheless, result in the formation of the similar 85S complexes. A detailed biochemical and cryo-EM investigation of the 85S complexes in PF and BF cells should shed light on this hypothetical scenario.

Mitochondrial translation in trypanosomatids is highly idiosyncratic, in line with virtually anything else in these organisms. The fact that mitochondrial translation is essential during the mammalian stage of the parasite’s life cycle indicates that the translation apparatus can be exploited as a selective target for the treatment of trypanosome- or leishmania-caused diseases.
